# Supplementary material for: Added value of anxiolytic benzodiazepines in predictive models on severe delirium in patients with acute decompensated heart failure: A retrospective analysis
Source: PLoS One. 2021 Apr 22;16(4):e0250372. doi: 10.1371/journal.pone.0250372 (PMC8062069; doi:10.1371/journal.pone.0250372)
Supplement: Attachment — Submitted filename: Response_to_Reviewers.docx [file pone.0250372.s002.docx]

Journal comments:

When submitting your revision, we need you to address these additional requirements.

**Comment 1:** Please ensure that your manuscript meets PLOS ONE's style requirements, including those for file naming. The PLOS ONE style templates can be found at

https://journals.plos.org/plosone/s/file?id=wjVg/PLOSOne_formatting_sample_main_body.pdf and

<https://journals.plos.org/plosone/s/file?id=ba62/PLOSOne_formatting_sample_title_authors_affiliations.pdf>

Response: Thank you for this comment. We have ensured that all PLOS ONE style requirements have been followed, including those for file naming.

**Comment 2:** Please modify the title to ensure that it is meeting PLOS’ guidelines (https://journals.plos.org/plosone/s/submission-guidelines#loc-title). In particular, the title should be "specific, descriptive, concise, and comprehensible to readers outside the field", and in this case the title may be misleadingly implying that your study is an experimental one, rather than an observational one.

**Response:** Thank you for this comment. We have modified the title to “Added value of anxiolytic benzodiazepines in predictive models on severe delirium in patients with acute decompensated heart failure: a retrospective analysis” accordingly.

**Comment 3:** We note that you have indicated that data from this study are available upon request. PLOS only allows data to be available upon request if there are legal or ethical restrictions on sharing data publicly. For information on unacceptable data access restrictions, please see http://journals.plos.org/plosone/s/data-availability#loc-unacceptable-data-access-restrictions.

**Response:** Thank you for this comment. We have now obtained permission from the Medical Research of Kochi Medical School’s institutional review board to publish all the study data. Therefore, we have provided the data as Supplementary Information files and changed the data availability statement.

Reviewers' comments:

**Response:** Thank you for your positive comments. We have thoroughly reviewed the whole manuscript and responded to all the comments carefully. Our point-by-point responses are listed below. The revised portions of the manuscript are provided as Tracked Changes.

Review Comments to the Author:

Reviewer #1: This study is aimed to evaluate the association of anxiolytic benzodiazepines use with delirium and their additive predictive value in models based on conventional risk factors. Data derived from a single-center, retrospective study, that enrolled 650 patients treated for ADHF. Fifty-nine patients (9.1%) had delirium. In multivariate analysis, anxiolytic benzodiazepines, mechanical ventilation or noninvasive positive pressure ventilation, depression, intensive care or high care unit admission, male sex, and age were independently associated with severe delirium. The predictive model that included anxiolytic benzodiazepines had a significantly better discriminatory ability for the incidence of severe delirium than the conventional model.

The study is of great interest, even if data derived from a single-center study, results confirmed also in HF patients the use of anxiolytic benzodiazepines as risk factor for severe delirium. Table 1 and 2 should be better presented.

**Response:** Thank you for your positive feedback and comment. It would appear that Tables 1 and 2 become misaligned when the document is converted to PDF at the time of submission. We have ensured that this mistake is not repeated when resubmitting our manuscript.

Reviewer #2: The authors retrospectively studied if delirium in patients with acute decompensated heart failure (ADHF) was associated with poor clinical outcomes in 650 patients treated for ADHF. Fifty-nine patients (9.1%) had delirium. In multivariate analysis, anxiolytic benzodiazepines [odds ratio (OR): 6.4, 95% confidence interval (CI): 2.8–15], mechanical ventilation or noninvasive positive pressure ventilation (OR: 6.0, 95% CI: 2.9–12), depression (OR: 3.2, 95% CI: 1.5–6.5), intensive care or high care unit admission (OR: 2.9, 95% CI: 1.5–5.6), male sex (OR: 2.0, 95% CI: 1–3.7), and age (OR: 1.1, 95% CI: 1–1.1) were independently associated with severe delirium. The predictive model that included anxiolytic benzodiazepines had a significantly better discriminatory ability for the incidence of severe delirium than the conventional model.

The manuscript is interesting and topic. However, I have some concerns about the precipitating factors for ADHF considered. Please see and discuss Testa G et al. Precipitating factors in younger and older adults with decompensated chronic heart failure: are they different? J Am Geriatr Soc. 2013 Oct;61(10):1827-8.

**Response:**

Thank you for your positive feedback and suggestion. We have added discussion points on the precipitating factors for ADHF and referenced Testa G et al. Precipitating factors in younger and older adults with decompensated chronic heart failure: are they different? J Am Geriatr Soc. 2013 Oct;61(10):1827-8 accordingly. The limitation below about the differ in younger and older adults was added in the discussion section and references.

Discussion (page 17, lines 220)

“In this study, we included potential risk factors for delirium as covariates during the regression analysis. However, delirium is more common in the elderly and their precipitating factors for ADHF are distinct from those in younger patients. Thus, future studies should focus on elucidating whether elderly-specific precipitating factors for ADHF, including infection and arrhythmia [44], could also be risk factors for delirium.”

References (page 24, lines 342)

(44) Gianluca T, David DM, Francesco C, Gaetano, Daniele D'A, Gianluigi G, et al. Precipitating factors in younger and older adults with decompensated chronic heart failure: are they different?. J Am Geriatr Soc. 2013;61: 1827-1828.”
